# Frailty Index and Cardiovascular Disease among Middle-Aged and Older Chinese Adults: A Nationally Representative Cross-Sectional and Follow-Up Study

**DOI:** 10.3390/jcdd9070228

**Published:** 2022-07-18

**Authors:** Xinyao Liu, Guolin Dai, Qile He, Hao Ma, Hongpu Hu

**Affiliations:** Institute of Medical Information/Medical Library, Chinese Academy of Medical Sciences & Peking Union Medical College, Beijing 100020, China; liu.xinyao@imicams.ac.cn (X.L.); dai.guolin@imicams.ac.cn (G.D.); he.qile@imicams.ac.cn (Q.H.); ma_hao@imicams.ac.cn (H.M.)

**Keywords:** frailty, cardiovascular disease, China, cross-sectional study, cohort study

## Abstract

Evidence for the association between the frailty index and cardiovascular disease (CVD) is inconclusive, and this association has not been evaluated in Chinese adults. We aim to examine the association between the frailty index and CVD among middle-aged and older Chinese adults. We conducted cross-sectional and cohort analyses using nationally representative data from the China Health and Retirement Longitudinal Study (CHARLS). From 2011 to 2018, 17,708 participants aged 45 years and older were included in the CHARLS. The primary outcome was CVD events (composite of heart disease and stroke). Multivariable adjusted logistic regression and Cox proportional hazards models were used to estimate the association between the frailty index and CVD in cross-sectional and follow-up studies, respectively. A restricted cubic spline model was used to characterize dose–response relationships. A total of 16,293 and 13,580 participants aged 45 years and older were included in the cross-sectional and cohort analyses, respectively. In the cross-sectional study, the prevalence of CVD in robust, pre-frailty and frailty was 7.83%, 18.70% and 32.39%, respectively. After multivariable adjustment, pre-frailty and frailty were associated with CVD; ORs were 2.54 (95% confidence interval [CI], 2.28–2.84) and 4.76 (95% CI, 4.10–5.52), respectively. During the 7 years of follow-up, 2122 participants without previous CVD developed incident CVD; pre-frailty and frailty were associated with increased risk of CVD events; HRs were 1.53 (95% CI, 1.39–1.68) and 2.17 (95% CI, 1.88–2.50), respectively. Furthermore, a stronger association of the frailty index with CVD was observed in participants aged <55, men, rural community-dwellers, BMI ≥ 25, without hypertension, diabetes or dyslipidemia. A clear nonlinear dose–response pattern between the frailty index and CVD was widely observed (*p* < 0.001 for nonlinearity), the frailty index was above 0.08, and the hazard ratio per standard deviation was 1.18 (95% CI 1.13–1.25). We observed the association between the frailty index and CVD among middle-aged and elderly adults in China, independent of chronological age and other CVD risk factors. Our findings are important for prevention strategies aimed at reducing the growing burden of CVD in older adults.

## 1. Introduction

Cardiovascular disease (CVD) is the leading cause of death in China [1]. Aging is a major driver of increased cardiovascular deaths, accounting for 64% [2]. In a progressively aging population, the potency of traditional risk factors to estimate the risk of CVD may decline. It has been suggested in recent years that biological age may be a more accurate predictor of risk of adverse outcomes than chronological age [3,4].

The frailty index, which measures frailty, is an emerging indicator of biological age [3,5]. Frailty, an important geriatric syndrome, is characterized by a cumulative decline in function across multiple physiological systems, and an increased vulnerability to stressors [5,6,7]. Frailty has been shown to be associated with increased mortality, hospitalization, falls, fracture and disability in cohorts of elderly people [3,8,9,10,11]. Moreover, previous studies have shown that the frailty index is a better predictor of adverse outcomes than DNA methylation among the elderly population [3,4,12].

However, limited studies have assessed the relationship between the frailty index and the risk of overall CVD events, and existing conclusions are rather controversial. Some studies showed that the frailty index is a predictor of CVD events [12,13], while other studies argued that the frailty index was not significantly associated with CVD events [14]. Discrepancies may be partly explained by differences in characteristic of population, sample size and follow-up time. In addition, to our knowledge, the association between the frailty index and CVD events has not been evaluated among Chinese adults.

We aimed to investigate the association between the frailty index and CVD among middle-aged and older Chinese adults, and compared the strength of such associations across different subgroups of age, gender, residence and traditional risk factors of CVD, by using nationally representative data from the China Health and Retirement Longitudinal Study (CHARLS).

## 2. Materials and Methods

### 2.1. Study Design and Participants

This study implemented cross-sectional analysis and cohort study design using data from CHARLS. CHARLS is a nationally representative cohort of community-dwelling adults aged 45 years and older in China, and is designed to collect information on socioeconomic status and individual health status. The baseline survey randomly selected 17,708 participants from 450 urban communities and rural areas within 28 provinces between June 2011 and March 2012 via multistage probability sampling method. Participants were followed every 2 or 3 years. The detailed study design for CHARLS has been previously reported [15]. CHARLS is approved by the Research Ethics Committee of Peking University (IRB00001052–11015). Written informed consent was obtained from all participants or their legal representatives. This study was reported following the Strengthening the Reporting of Observational Studies in Epidemiology (STROBE) guideline [16].

**Figure 1:**
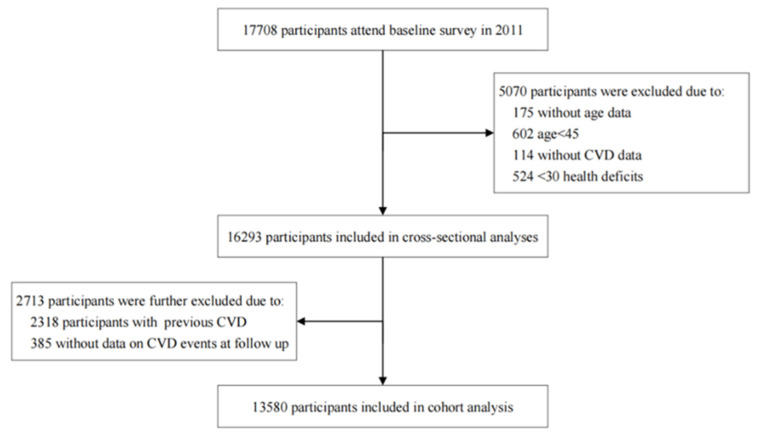
Flowchart of study participants.

For cross-sectional analysis, survey data from CHARLS 2011 were used. Participants were excluded for the following criteria: (1) individuals aged <45 years old; (2) <30 deficits; (3) no CVD events data. For the cohort analysis, data from CHARLS 2011, 2013, 2015 and 2018 were used, and participants with prior CVD at baseline or without data on CVD events at follow-up were further excluded. Finally, A total of 16,293 and 13,580 participants aged over 45 years old were included in the cross-sectional and longitudinal analyses, respectively. The selection process of individuals included in the study is shown in Figure 1.

### 2.2. Measurement

#### 2.2.1. Frailty Index

The frailty index was conducted following a standard procedure [17]. Health deficits were included in the frailty index if (1) they were associated with health status; (2) the prevalence of health deficits accumulated with age, but did not saturate in middle-age; (3) the health deficits had a prevalence >1%. Based on previous studies [18,19,20,21] and CHARLS data catalog, activity of daily living (ADL) disabilities and instrumental activity of daily living (IADL) disabilities (11 items), physical function limitations (9 items), chronic diseases (9 items), mental health indicators (5 items) and subjective functioning (self-rated health) were included. Four traditional risk factors associated with CVD, including hypertension, diabetes mellitus, dyslipidemia and body mass index (BMI), were not included. Each health deficit was recoded as 0.00 (no deficit) −1.00 (all items exhibit deficits) interval. The frailty index list is shown in Table S1.

We included participants with ≥30 items of health deficits given that 30 items is a minimum threshold in various universal standards of the deficit list [17]. For each individual, the frailty index was calculated by summing all health deficits and dividing by the total number of health deficits. In accordance with previous studies, we classified the continuous frailty index into robust (frailty index ≤0.10), prefrail (frailty index >0.10 to <0.25), and frail (frailty index ≥0.25) [3].

#### 2.2.2. Outcomes

The primary outcome in this study was CVD events, a composite of heart disease and stroke. With reference to previous studies [22,23], CVD events were identified by the following questions: “Have you been told by a doctor that you have been diagnosed with heart disease (including heart attack, angina, coronary heart disease, heart failure, or other heart problems)?” or “Have you been told by a doctor that you have been diagnosed with a stroke?”. The secondary outcomes were separate components of the primary outcome, including heart disease and stroke.

#### 2.2.3. Covariates

Based on previous studies, we controlled potential confounding covariates in the analysis [23,24], including age; gender (men/women); residential area (rural/urban); marital status (married/other); education level (no formal education/elementary school/junior high school or above); smoking status (current smoker/non-current smoker); drinking status (drinker/non-drinker); BMI; blood pressure; self-reported physician-diagnosed chronic diseases including hypertension, diabetes mellitus and dyslipidemia; and medication status of corresponding disease (yes/no). BMI was calculated as weight in kg/height in m^2^.

In the cross-sectional analysis, a subgroup of 10,803 participants underwent biomarker measurements including fasting blood glucose (FBG), glycated hemoglobin (HbA1c), total cholesterol (TC), triglycerides (TG), low-density lipoprotein cholesterol (LDL-c), high-density lipoprotein cholesterol (HDL-c) and serum creatinine. In the cohort analysis, a subgroup of 8971 participants underwent biomarker measurements. The estimated glomerular filtration rate (eGFR) was calculated according to the creatinine equation stipulated by the Chronic Kidney Disease Epidemiology Collaboration in 2009 [25]. The detailed information of biomarker sample collection and analysis has been reported in previous studies [26].

### 2.3. Statistical Analysis

First, the covariate characteristics of participants with different frailty status were summarized. Distributions of characteristics were presented using frequencies (%) for categorical variables, mean (standard deviation, SD) or median (interquartile range, IQR) for continuous variables. We used χ^2^ test for categorical variables, as appropriate, and ANOVA or Kruskal–Wallis for continuous variables to compare covariates’ characteristics in different frailty statuses. Bonferroni correction was applied for multiple comparisons. A chain equation model was used to account for missing data on covariates with 10 multiple imputations for covariates missing.

Next, for cross-sectional analysis, the association between the frailty index and CVD was estimated using odds ratios (ORs) and 95% confidence intervals (CIs) calculated from logistic regression model.

Then, for longitudinal analysis, Cox proportional hazards models with hazard ratios (HRs) and 95% CIs were used to examine the associations of the frailty index with CVD events. The Schoenfeld residuals were used to test the proportional hazards assumption. The endpoint was the first occurrence of CVD events. Follow-up started from baseline to the date of the first CVD events, death, loss to follow-up or the end of study (November 2018), whichever occurred first. Date of CVD diagnosis or death was defined as between the date of reported occurrence of CVD or death and the date of the last interview [22,23]. The date of the last interview was censored if the participant was lost to follow-up. For cross-sectional and longitudinal analysis, the frailty index was first examined as a categorical variable. The frailty index was also examined as continuous, with a HR indicating a 0.1 increment in the risk of developing a CVD event. We fitted three models: model 1 was adjusted for age and gender only; model 2 was additionally adjusted for educational level, marital status, residence, smoking status, drinking status, hypertension, diabetes mellitus, dyslipidemia, and use of antihypertensive medications, antidiabetic medications and lipid-lowering therapy; and model 3 was further adjusted for BMI, systolic blood pressure (SBP) and diastolic blood pressure (DBP). Moreover, we performed a restricted cubic spline with three knots at the 10th, 50th, and 90th centiles to explore the potential nonlinear association between the frailty index and CVD.

Additionally, we estimated the interaction of the frailty index with age, sex, BMI, residence, hypertension, diabetes mellitus and dyslipidemia to determine the risk of CVD by including interaction terms in the logistic regression models and Cox proportional hazards models. Once statistically significant interaction was observed, the subgroup analysis was conducted. Hypertension was defined as SBP ≥ 140 mmHg, DBP ≥ 90 mmHg, self-reported hypertension diagnosed by a physician, or on antihypertensive medication [23]. Diabetes mellitus was defined as FPG ≥ 126 mg/dL (7.0 mmol/L), HbA1c ≥ 6.5%, self-reported diabetes mellitus diagnosed by a physician or on antidiabetes medication [27,28]. Dyslipidemia was determined whether any of the following criteria were met: TC ≥ 240 mg/dL, TG ≥ 150 mg/dL, LDL-c ≥ 160 mg/dL, HDL-c < 40 mg/dL, self-reported dyslipidemia diagnosed by a physician or on lipid-lowering medication [23].

Finally, we performed the following three sensitivity analyses to assess the robustness of the findings: (1) We additionally adjusted for biomarkers, including FBG, HbA1c, TC, TG, LDL-c, HDL-c and eGFR. (2) We assessed the effect of death as a competing risk for CVD using the Fine and Gray competing risk model. Because the frailty index was associated with mortality, if people with higher frailty index died before developing CVD, then the actual number of CVD events will be reduced, thereby affecting the probability of CVD events. (3) The main results of the cross-sectional and cohort studies analyses were repeated in the complete dataset with no missing covariates.

Sampling weights were not used in all analyses because several studies have shown similar results with and without weighting [29,30]. All analyses were conducted in SAS version 9.4 or R version 4.1.2. All *p* values were two-sided, and *p* < 0.05 was considered significant.

## 3. Results

### 3.1. Participant Characteristics

A total of 16,293 and 13,580 participants were included in cross-sectional and cohort analyses, respectively. Detailed characteristics of the participants in cross-sectional and cohort analyses are presented in Table S2 and Table 1, respectively. Of the 13,580 participants in the longitudinal analysis, the mean age was 58.43 (SD 9.54) and 6787 (49.98%) were male. The prevalence of pre-frailty and frailty was 33.74% (4583/13,580), 7.95% (1080/13,580), respectively. The median frailty index was 0.08 (IQR 0.04–0.15) in all population. Compared with those who in other frailty status, participants with robust were more likely to be younger, women, educated and live in urban areas, and with lower prevalence of hypertension, diabetes mellitus, dyslipidemia and corresponding medication history.

### 3.2. Association between the Frailty Index and CVD in Cross-Sectional Analysis

Table S3 shows the association between the frailty index and CVD in the cross-sectional analysis. The prevalence of CVD in robust, pre-frailty and frailty was 7.83%, 18.70% and 32.39%, respectively. After further adjustments for all the included covariates in model 3, both pre-frailty and frailty were significantly associated with CVD, with ORs of 2.54 (95% CI, 2.28–2.84), 4.76 (95% CI, 4.10–5.52), respectively; OR for prevalence of CVD per 0.1 increment in the frailty index was 1.64 (95% CI, 1.57–1.71).

### 3.3. Association between the Frailty Index and CVD Events in Cohort Analysis

Table 2 shows the association between the frailty index and CVD in the cohort analysis. All models for event CVD satisfied the proportional hazards assumption. During a follow-up period of up to 7 years, 2122 CVD events occurred in 13,580 participants with no history of CVD at baseline. Taking the robust as reference, pre-frailty and frailty were associated with high risk of CVD events in model 3, with HRs of 1.53 (95% CI, 1.39–1.68), 2.17 (95% CI, 1.88–2.50), respectively. Each 0.1 increment in the frailty index was associated with a 29% increase in risk of CVD events (HR, 1.29, [95% CI, 1.24–1.34)].

**Figure 2:**
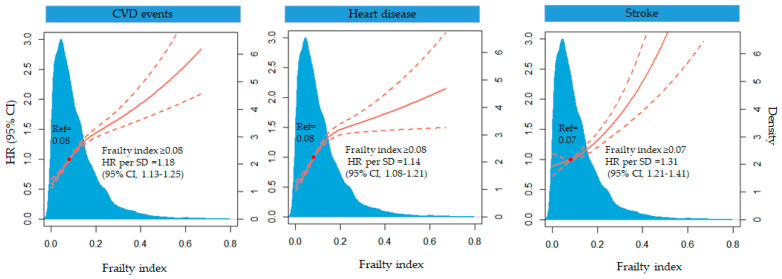
Multivariable-adjusted HR for CVD, heart disease and stroke events according to levels of frailty index. The solid red line is the multivariate adjusted hazard ratios, and the dashed red line is the 95% CI from a restricted cubic spline model with three knots at the 10th, 50th and 90th centiles. Model was adjusted for age, gender, educational level, marital status, residence, smoking status, drinking status, hypertension, diabetes mellitus, dyslipidemia, use of antihypertensive medications, antidiabetic medications and lipid-lowering therapy, BMI, SBP and DBP.

In the restricted cubic spline model, CVD events increased relatively stably until the frailty index reached 0.08 and then increased rapidly (*p* <0.0001 for nonlinearity). When the frailty index was above 0.08, the HR per standard deviation was 1.18 (95% CI 1.13–1.25) (Figure 2).

### 3.4. Subgroup Analysis

**Figure 3:**
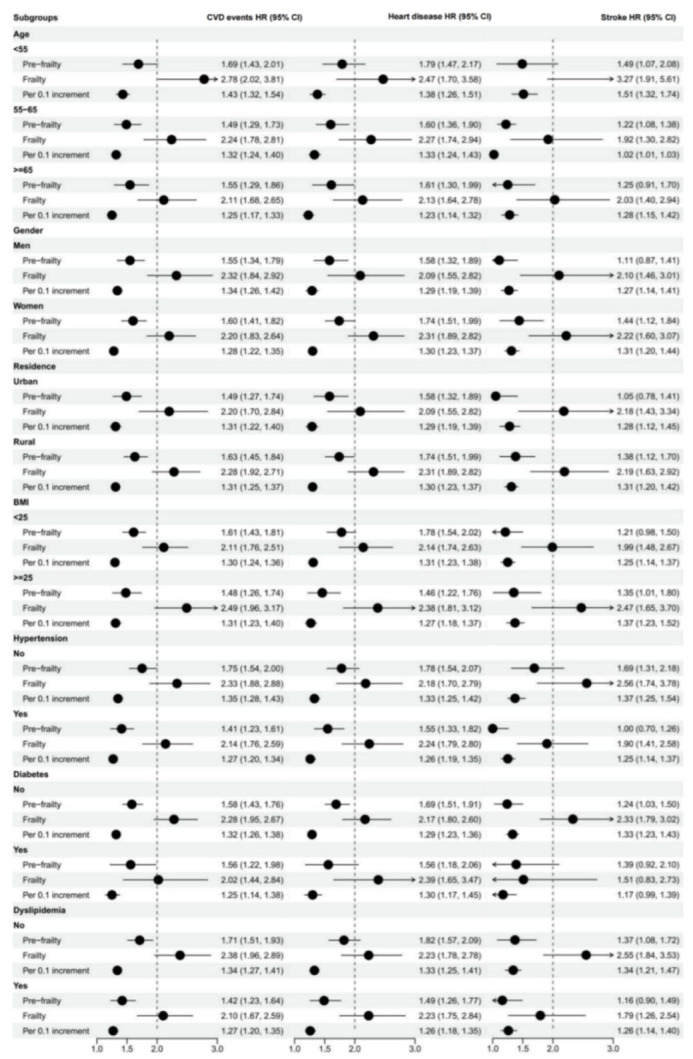
Longitudinal association between the frailty index and CVD events stratified by age, gender, residence, BMI, and chronic disease status. Model was adjusted for age, gender, educational level, marital status, residence, smoking status, drinking status, hypertension, diabetes mellitus, dyslipidemia and BMI.

Figure S1 and Figure 3 show the results of subgroup analyses in cross-sectional and cohort analyses, respectively. In longitudinal analysis, statistically significant interactions between the frailty index and all subgroups were observed (*p* for interaction <0.0001, after controlling potential confounders). We found significant positive associations between the frailty index and CVD in all subgroups, with the risk of frailty for CVD events being more evident in participants aged <55, men, rural community-dwellers, BMI ≥ 25, and without hypertension, diabetes or dyslipidemia.

Similar associations were observed in the analysis on the secondary outcomes.

### 3.5. Sensitivity Analysis

The results were basically unchanged after further adjustment for metabolic biomarkers (Tables S4 and S5). Results did not materially change when considering death as a competing CVD event compared with estimates in the primary analysis (Table S6). Models using the complete dataset (with all missing covariates removed) produced similar results to the main analysis (Tables S7 and S8). The results of the sensitivity analysis indicated the robustness of our findings.

## 4. Discussion

To our knowledge, this study is the first cross-sectional and cohort analysis of a large longitudinal and nationally representative cohort to determine the association between the frailty index and CVD risk in a Chinese middle-aged and elderly population. Results showed that the frailty index which does not include any traditional CVD risk factors, is associated with CVD events in both cross-sectional and cohort analyses, and this association is independent of chronological age and several traditional risk factors with CVD. Notably, the strength of the association was stronger in participants without hypertension, diabetes mellitus, dyslipidemia.

Interestingly, we also found that the strength of frailty index to predict CVD events decreased with age. This suggests that frailty assessment in midlife may help prevent late CVD development. Similarly, several prospective studies showed that the strength of the frailty index to predict mortality events decreased with age [3,31,32]. Different from previous studies [31,32], we found that compared with women, men had a higher prevalence of frailty and a stronger association between the frailty index and CVD. Furthermore, few studies have investigated differences in associations between the frailty index and CVD in residence and BMI level. Our study raises the possibility that the strength of the association was stronger in rural areas and individuals with BMI ≥ 25, though further investigations should be undertaken to explore the detailed relationship and their underlying mechanisms.

Several studies have explored the association of the frailty index with CVD, but no consistent conclusions have been reached. A cohort study from the English Longitudinal Study of Ageing with 5294 participants (mean age, 71.2 years; 44.9% men) showed that the frailty index with 40-item health deficits was not associated with CVD events over 7 years [14]. However, a pooled analysis of 154,000 older adults (mean age, 70.8 years; 63% men) from 14 multicenter clinical trials with a median follow-up of 3.2 years found that the frailty index with a 26-item excluded CVD risk factors was associated with CVD events [12]. Nonetheless, the study included participants with CVD. A study of 2195 adults (mean age, 47 years; 48% men) without coronary heart disease from the Nova Scotia Health Survey found that a 17-item frailty index without traditional risk factors was associated with incident CVD events over 10 years [13]. However, the study only adjusted for age and sex.

Our study further confirmed that the frailty index can be used as a predictor of CVD events by adjusting for conventional CVD risk factors more comprehensively, even in individuals without CVD and its relative risks. We also observed a nonlinear association that the frailty index greater than 0.08 was associated with a higher risk of CVD events, extending the above findings. The frailty phenotype, another common assessment of frailty, is thought to provide information complementary to the frailty index [33]. Recent studies have shown that the frailty phenotype is also associated with a higher risk of CVD events [34,35]. The observed relationships between frailty and CVD may partly be explained by several potential biological mechanisms and their interplay, involving inflammation, insulin resistance, and coagulation problems [36,37]. Another possible explanation is the effects of common adverse lifestyle factors such as smoking, lack of physical activities, unhealthy dietary patterns and traditional CVD risk factors such as high blood pressure, diabetes, low HDL cholesterol and obesity [38].

The present findings have important implications for developing targeted policies in public health, including implementing frailty screening and disease identification in vulnerable groups and providing health management, precision medical services and interventions and health education for frail older adults. These suggested measures might promote the prevention of CVD and reduce the burden on both individual expenditure and national health systems to a great degree, in view of the current high prevalence of frailty in Chinese adults aged over 60 years (7.0% to 26.9%) [3,39,40,41,42,43,44,45,46] and in the elderly population with CVD (60%) [47,48]. However, whether the frailty index in middle-aged individuals is representative of aging has not been established, and more research is needed to replicate our findings.

This study has several limitations. First, data on specific CVD were not available, and self-reported physician diagnoses of CVD may be subject to recall bias. However, CHARLS data were collected by professionally trained investigators to reduce such biases. Second, cognitive function was not included in the construction of the frailty index due to disproportionately missing data, which may lead to a bias in estimating the prevalence of frailty from those reported by previous studies. However, the study showed that the bias in the findings was less related to the items included and depended more on the number of health deficits. Third, although our study controlled as many traditional CVD risk factors as possible, other potential confounders such as dietary habits, physical activities and muscle strength could not be completely ruled out. Finally, our data are limited to 7 years, so it would be advisable to follow up longer and further analyze the effect of changes in frailty-index trajectories on CVD.

## 5. Conclusions

In conclusion, our study shows that in middle-aged and older Chinese adults, the frailty index is significantly associated with CVD. Our current findings preliminarily highlight the importance of frailty screening and effective interventions for improving primary prevention of CVD in middle-aged and older adults.

## Figures and Tables

**Table 1:** Baseline characteristics of study population by frailty status in longitudinal analysis.

Characteristic	Total (N = 13,580)	Robust (N = 7917)	Pre-Frailty (N = 4583)	Frailty (N = 1080)	*p* Value
Age (mean ± SD)	58.43 ± 9.54	56.53 ± 8.62	60.01 ± 9.61	65.65 ± 10.86	<0.0001
Sex, Male, n (%)	6787 (49.98)	3489 (44.07)	2628 (57.34) *	670 (62.04) *^,†^	<0.0001
Education level, n (%)					<0.0001
No formal education (Illiterate)	3713 (27.34)	1555 (19.64)	1594 (34.78) *	564 (52.22) *^,†^	
Elementary school	5273 (38.83)	2914 (36.81)	1975 (43.09) *	384 (35.56) *^,†^	
Junior high school or above	4594 (33.83)	3448 (43.55)	1014 (22.13) *	132 (12.22) *^,†^	
Married, n (%)	11,002 (81.02)	6586 (83.19)	3639 (79.40) *	777 (71.94) *^,†^	<0.0001
Residence, rural, n (%)	8322 (61.28)	4355 (55.01)	3166 (69.08) *	801 (74.17) *^,†^	<0.0001
Current smoker, n (%) ^a^	4339 (32.14)	2755 (35.08)	1337 (29.25) *	247 (23.00) *^,†^	<0.0001
Drinker, n (%)	4773 (35.15)	3180 (40.17)	1371 (29.91) *	222 (20.56) *^,†^	<0.0001
Comorbidities ^a^					
Hypertension, n (%)	2917 (21.52)	1468 (18.56)	1113 (24.34) *	336 (31.26) *^,†^	<0.0001
Diabetes mellitus, n (%)	663 (4.89)	323 (4.08)	254 (5.56) *	86 (8.01) *^,†^	<0.0001
Dyslipidemia, n (%)	1033 (7.65)	558 (7.07)	381 (8.36) *	94 (8.88) *^,†^	0.0039
History of medication use, n (%) ^a^					
Antihypertensive medications	2114 (15.60)	1029 (13.02)	817 (17.87) *	268 (24.98) *^,†^	<0.0001
Antidiabetic medications	430 (3.17)	196 (2.48)	168 (3.68) *	66 (6.16) *^,†^	<0.0001
Lipid-lowering therapy	470 (3.48)	231 (2.93)	185 (4.06) *	54 (5.10) *^,†^	<0.0001
Height, m (mean ± SD)	1.58 ± 0.09	1.60 ± 0.08	1.57 ± 0.08 *	1.54 ± 0.09 *^,†^	<0.0001
Weight, kg (mean ± SD)	58.46 ± 11.67	60.26 ± 11.63	56.70 ± 11.18 *	53.78 ± 11.67 *^,†^	<0.0001
BMI, Kg/m^2^ (mean ± SD) ^a^	23.30 ± 3.86	23.58 ± 3.79	23.03 ± 3.92 *	22.56 ± 3.95 *^,†^	<0.0001
SBP (mean ± SD) ^a^	129.00 ± 21.13	128.43 ± 20.26	128.94 ± 21.64	133.01 ± 24.02 *^,†^	<0.0001
DBP (mean ± SD) ^a^	76.67 ± 12.78	77.16 ± 12.74	75.85 ± 12.67 *	77.01 ± 13.31	<0.0001
Biomarkers ^b,c^					
FBG, mg/dL	102.24 (94.32, 112.86)	102.24 (94.32, 113.04)	101.70 (93.78, 112.14)	102.60 (94.68, 116.10) ^†^	0.0174
HbA1c, %	5.1 (4.9, 5.4)	5.1 (4.9, 5.4)	5.1 (4.9, 5.4)	5.2 (4.9, 5.5)	0.0093
TC mg/dL	192.63 ± 37.73	192.54 ± 38.11	193.16 ± 37.03	190.98 ± 38.06	0.3595
TG, mg/dL	104.43 (74.34, 152.22)	104.43 (74.34, 153.88)	103.55 (74.34, 147.80)	106.20 (77.00, 157.53)	0.2979
LDL-c, mg/dL	115.88 ± 34.62	115.88 ± 35.107	116.38 ± 34.06	113.38 ± 33.87	0.1055
HDL-c, mg/dL	51.43 ± 15.32	50.76 ± 15.09	52.45 ± 15.38 *	51.61 ± 16.36	<0.0001
eGFR, mL/min/1.73 m^2^	90.16 (71.50, 102.34)	91.88 (75.45, 103.68)	88.40 (68.67, 101.11) *	84.28 (61.33, 98.44) *^,†^	<0.0001
Frailty index, mean (IQR)	0.08 (0.04, 0.15)	0.05 (0.02, 0.07)	0.15 (0.12, 0.19) *	0.32 (0.28, 0.39) *^,†^	<0.0001

Abbreviation: BMI, body mass index; SBP, systolic blood pressure; DBP, diastolic blood pressure; FBG, fasting blood glucose; HbA1c, glycated hemoglobin; TC, total cholesterol; TG, triglycerides. LDL-c, low-density lipoprotein cholesterol; HDL-c, high-density lipoprotein cholesterol; eGFR, estimated glomerular filtration rate. ^a^ Missing data: 81 for smoking status, 24 for hypertension, 29 for diabetes mellitus, 74 for dyslipidemia, 28 for hypertension medications, 31 for diabetes medications, 77 for lipid-lowering therapy, 2779 for BMI, 2721 for SBP, 2694 for DBP. ^b^ Measured in subpopulation of 8971 participants. ^c^ Data shown as median (IQR) or mean ± SD. * Different from the robust. ^†^ Different from the pre-frailty.

**Table 2:** Longitudinal association between the frailty index and CVD events.

Outcome	Case/N	Incidence per 1000 Person-Years	Model 1 HR (95% CI)	Model 2 HR (95% CI)	Model 3 HR (95% CI)
CVD events					
Robust	954/7917	22.47	Reference	Reference	Reference
Pre-frailty	890/4583	36.52	1.53 (1.39, 1.68) ***	1.51 (1.37, 1.66) ***	1.53 (1.39, 1.68) ***
Frailty	278/1080	62.21	2.19 (1.91, 2.52) ***	2.13 (1.85, 2.46) ***	2.17 (1.88, 2.50) ***
Per 0.1 increment			1.30 (1.25, 1.35) ***	1.28 (1.23, 1.33) ***	1.29 (1.24, 1.34) ***
Heart disease					
Robust	707/7917	16.43	Reference	Reference	Reference
Pre-frailty	701/4583	28.30	1.61 (1.44, 1.79) ***	1.60 (1.44, 1.79) ***	1.62 (1.45, 1.81) ***
Frailty	209/1080	41.60	2.14 (1.82, 2.52) ***	2.19 (1.80, 2.50) ***	2.16 (1.83, 2.56) ***
Per 0.1 increment			1.28 (1.23, 1.34) ***	1.27 (1.21, 1.33) ***	1.28 (1.22, 1.34) ***
Stroke					
Robust	322/7917	7.25	Reference	Reference	Reference
Pre-frailty	256/4583	9.71	1.23 (1.07, 1.50) **	1.20 (1.01, 1.43) *	1.23 (1.03, 1.46) *
Frailty	102/1080	18.38	2.26 (1.79, 2.86) ***	2.01 (1.59, 2.53) ***	2.06 (1.62, 2.63) ***
Per 0.1 increment			1.31 (1.23, 1.41) ***	1.27 (1.18, 1.36) ***	1.28 (1.20, 1.37) ***

Statistically significant at * *p* < 0.05, ** *p* < 0.01, and *** *p <* 0.001. Model 1 was adjusted age and gender. Model 2 was additionally adjusted educational level, marital status, residence, smoking status, drinking status, hypertension, diabetes mellitus, dyslipidemia, and use of antihypertensive medications, antidiabetic medications and lipid-lowering therapy. Model 3 was additionally adjusted BMI, SBP and DBP.

## Data Availability

CHARLS data are available via the website: https://charls.charlsdata.com/pages/data/111/zh-cn.html (accessed on 1 December 2021).

## Supplementary Materials

The following supporting information can be downloaded at: https://www.mdpi.com/article/10.3390/jcdd9070228/s1, Table S1: List of health-deficit items included in the frailty index; Table S2: Baseline characteristics of 16,293 participants by frailty status in the cross-sectional analysis; Table S3: Cross-sectional association between the frailty index and CVD; Table S4: Cross-sectional association between the frailty index and CVD in subpopulations of 10,803 participants who underwent biomarker measurements; Table S5: Longitudinal association between the frailty index and CVD in subpopulations of 8971 participants who underwent biomarker measurements; Table S6: Longitudinal association between the frailty index and CVD events by competing risk analysis; Table S7: Cross-sectional association between the frailty index and CVD in complete dataset; Table S8: Longitudinal association between the frailty index and CVD events in complete dataset; Figure S1: Cross-sectional association between the frailty index and CVD stratified by age, gender, residence, BMI and chronic disease status.
