# Supplementary material for: Frailty Index and Cardiovascular Disease among Middle-Aged and Older Chinese Adults: A Nationally Representative Cross-Sectional and Follow-Up Study
Source: J Cardiovasc Dev Dis. 2022 Jul 18;9(7):228. doi: 10.3390/jcdd9070228 (PMC9319589; doi:10.3390/jcdd9070228)
Supplement: Supplementary file 1 [file jcdd-09-00228-s001.zip › jcdd-1816311-supplementary.pdf]

Supplementary material

Table S1. List of health deficits items included in the frailty index

Table S2. Baseline characteristics of 16,293 participants by frailty status in the cross-sectional analysis

Table S3. Cross-sectional association between the frailty index and CVD

Table S4. Cross-sectional association between the frailty index and CVD in subpopulations of 10,803 participants underwent biomarkers measurements

Table S5. Longitudinal association between the frailty index and CVD in subpopulations of 8971 participants underwent biomarkers measurements

Table S6. Longitudinal association between the frailty index and CVD events by competing risk analysis

Table S7. Cross-sectional association between the frailty index and CVD in complete dataset

Table S8. Longitudinal association between the frailty index and CVD events in complete dataset

Figure S1. Cross-sectional association between the frailty index and CVD stratified by age, gender, residence, BMI, and chronic disease status

Table S1. List of health deficits items included in the frailty index.

Type of deficit	Item	Variables	Cut-off point
Activities of daily living	1	Bathing	No=0; a little =0.33; need help = 0.67; Yes=1
	2	Dressing	No=0; a little =0.33; need help = 0.67; Yes=1
	3	Use of toilet	No=0; a little =0.33; need help = 0.67; Yes=1
	4	Transferring	No=0; a little =0.33; need help = 0.67; Yes=1
	5	Continence	No=0; a little =0.33; need help = 0.67; Yes=1
	6	Eating	No=0; a little =0.33; need help = 0.67; Yes=1
Instrumental activities of daily living	7	Cooking	No=0; a little =0.33; need help = 0.67; Yes=1
	8	Shopping	No=0; a little =0.33; need help = 0.67; Yes=1
	9	Doing household	No=0; a little =0.33; need help = 0.67; Yes=1
	10	Taking medicine	No=0; a little =0.33; need help = 0.67; Yes=1
	11	Managing money	No=0; a little =0.33; need help = 0.67; Yes=1
Physical functional limitations	12	Lift a weight of 5 kg	No=0; a little =0.33; need help = 0.67; Yes=1
	13	Walking 1 km	No=0; a little =0.33; need help = 0.67; Yes=1
	14	Walking 100m	No=0; a little =0.33; need help = 0.67; Yes=1
	15	Stooping, kneeling, or crouching	No=0; a little =0.33; need help = 0.67; Yes=1
	16	Able to stand up from sitting	No=0; a little =0.33; need help = 0.67; Yes=1
	17	Able to pick up a coin from a table	No=0; a little =0.33; need help = 0.67; Yes=1
	18	Running or jogging about 1 km	No=0; a little =0.33; need help = 0.67; Yes=1
	19	Reaching or extend arms	No=0; a little =0.33; need help = 0.67; Yes=1
	20	Climbing several flights of stairs without resting	No=0; a little =0.33; need help = 0.67; Yes=1
Chronic disease	21	Chronic lung diseases (Chronic bronchitis, emphysema)	Yes=1, no=0
	22	Asthma	Yes=1, no=0

	23	Cancer	Yes=1, no=0
	24	Arthritis or Rheumatism	Yes=1, no=0
	25	Gastric or duodenal ulcer	Yes=1, no=1
	26	Kidney disease	Yes=1, no=1
	27	Liver disease	Yes=1, no=1
	28	Memory related disease (Dementia, brain atrophy, and Parkinson's disease)	Yes=1, no=0
	29	Emotional, nervous, or psychiatric problems	Yes=1, no=0
Mental health	30	Feel depressed	Always=1; often=0.33; sometimes=0.67; seldom or never=0
	31	Feel fearful	Always=1; often=0.33; sometimes=0.67; seldom or never=0
	32	Feel happy	Always=1; often=0.33; sometimes=0.67; seldom or never=0
	33	Feel everything was an effort	Always=1; often=0.33; sometimes=0.67; seldom or never=0
	34	Feel could not get "going"	Always=1; often=0.33; sometimes=0.67; seldom or never=0
Subjective functioning	35	Self-rated health	Very good=0; good=0.25; average=0.5; bad=0.75; very bad=1

Table S2. Baseline characteristics of 16,293 participants by frailty status in the cross-sectional analysis.

Characteristic	Total (N=16,293)	Robust (N=8791)	Pre-frailty (N=5844)	Frailty (N=1658)	*P* value
Age (mean ± SD)	58.95 ± 9.61	56.83 ± 8.73	60.34 ±9.59 *	65.26 ± 10.41 *†	<0.0001
Sex, Male, n (%)	7959 (48.85)	4913 (55.89)	2436 (41.68) *	610 (36.79) *†	<0.0001
Education level, n (%)					<0.0001
No formal education (Illiterate)	4437 (27.23)	1692 (19.25)	1933 (33.08) *	812 (48.97) *†	
Elementary school	6370 (39.10)	3237 (36.82)	2520 (43.12) *	613 (36.97) *†	
Junior high school or above	5486 (33.67)	3862 (43.93)	1391 (23.80) *	233 (14.05) *†	
Married, n (%)	13,158 (80.76)	7319 (83.26)	4620 (79.06) *	1219 (73.52) *†	<0.0001
Residence, rural, n (%)	9734 (59.74)	4723 (53.73)	3852 (65.91) *	1159 (69.90) *†	<0.0001
Current smoker, n (%) [a]	4984 (30.76)	3014 (34.56)	1620 (27.79) *	350 (21.21) *†	<0.0001
Drinker, n (%)	5465 (33.54)	3484 (39.63)	1667 (28.52) *	314 (18.94) *†	<0.0001
Comorbidities [a]					
Hypertension, n (%)	4252 (26.15)	1842 (20.97)	1754 (30.09) *	656 (39.73) *†	<0.0001
Diabetes, n (%)	1008 (6.20)	405 (4.61)	420 (7.21) *	183 (11.10) *†	<0.0001
Dyslipidemia, n (%)	1611 (9.95)	708 (8.08)	661 (11.39) *	242 (14.86) *†	<0.0001
History of medication use, n (%) [a]					
Antihypertensive medications	2406 (19.04)	970 (14.65)	1070 (22.26) *	366 (30.20) *†	<0.0001
Antidiabetic medications	516 (4.08)	179 (2.70)	238 (4.95) *	99 (8.17) *†	<0.0001
Lipid-lowering therapy	627 (4.96)	222 (3.35)	284 (5.91) *	121 (9.98) *†	<0.0001
Height, m (mean ± SD)	1.58 ± 0.09	1.60 ± 0.08	1.57 ± 0.08 *	1.54 ± 0.09 *†	<0.0001
Weight, kg (mean ± SD)	58.81 ± 11.86	60.51 ± 11.72	57.46 ± 11.58 *	55.09 ± 11.11 *†	<0.0001
BMI, Kg/m^2 (mean ± SD) [a]	23.45 ± 3.94	23.65 ±3.81	23.29 ± 4.05 *	23.02 ± 4.14 *	<0.0001

Continued

Table S2 continued					
Characteristic	Total (N=16,293)	Robust (N=8791)	Pre-frailty (N=5844)	Frailty (N=1658)	*P* value
SBP (mean ± SD) [a]	129.78 ± 21.50	128.86 ± 20.41	129.94 ± 22.10	133.80 ± 23.96 *†	<0.0001
DBP (mean ± SD) [a]	76.94 ± 12.90	77.32 ± 12.77	76.29 ± 12.86 *	77.52 ± 13.52	<0.0001
Biomarkers [b, c]					
FBG, mg/dL	102.42 (94.50,113.58)	102.60 (94.68, 113.40)	102.24 (93.96, 113.04)	103.14 (95.22, 116.64) *†	0.0066
HbA1c, %	5.1 (4.9, 5.4)	5.1 (4.9, 5.4)	5.1 (4.9, 5.5) *	5.2 (4.9, 5.5) *	<0.0001
TC, mg/dL	193.20 ± 38.28	192.80 ± 38.49	193.61 ± 37.67	193.69 ± 39.41	0.5288
TG, mg/dL	106.20 (75.23, 155.76)	105.32 (75.23, 156.65)	105.32 (75.23, 151.34)	113.28 (79.65, 165.50) *†	0.0015
LDL-c, mg/dL	116.37 ± 34.94	116.10 ± 35.23	116.91 ± 34.60	115.78 ± 34.70	0.4408
HDL-c, mg/dL	51.07 ± 15.33	50.60 ± 15.21	51.85 ± 15.31 *	50.60 ± 15.92 †	0.0002
eGFR, mL/min/1.73 m^2	90.52 (72.10, 102.86)	92.09 (75.90, 103.83)	89.31 (70.11, 102.40) *	86.13 (63.12, 99.47) *†	<0.0001
Frailty index, mean (IQR)	0.09 (0.04,0.16)	0.05 (0.02,0.07)	0.15 (0.12,0.19) *	0.33 (0.28,0.40) *†	<0.0001

Abbreviation: BMI, body mass index; SBP, systolic blood pressure; DBP, diastolic blood pressure; FBG, fasting blood glucose; HbA1c, glycated hemoglobin; TC, total cholesterol; TG, triglycerides. LDL-c, low-density lipoprotein cholesterol; HDL-c, high-density lipoprotein cholesterol; eGFR, estimated glomerular filtration rate.

[a] Missing data: 92 for smoking status, 30 for hypertension, 36 for diabetes mellitus, 104 for dyslipidemia, 34 for hypertension medications, 38 for diabetes medications, 113 for lipid-lowering therapy, 3404 for BMI, 3315 for SBP, 3284 for DBP.

[b] Measured in subpopulation of 10,803 participants.

[c] Data shown as median (IQR) or mean ± SD.

*Different from the robust. †Different from the pre-frailty.

Table S3. Cross-sectional association between the frailty index and CVD.

Outcome	Case/N	Model 1 OR (95% CI)	Model 2 OR (95% CI)	Model 3 OR (95% CI)
CVD events				
Robust	688/8791	Reference	Reference	Reference
Pre-frailty	1093/5844	2.47 (2.23, 2.75) ***	2.54 (2.27, 2.83) ***	2.54 (2.28, 2.84) ***
Frailty	537/1658	4.64 (4.05, 5.32) ***	4.74 (4.09, 5.50) ***	4.76 (4.10, 5.52) ***
Per 0.1 increment		1.63 (1.57, 1.69) ***	1.64 (1.57, 1.71) ***	1.64 (1.57, 1.71) ***
Heart disease				
Robust	611/8791	Reference	Reference	Reference
Pre-frailty	965/5844	2.40 (2.15, 2.68) ***	2.48 (2.21, 2.79) ***	2.49 (2.22, 2.80) ***
Frailty	421/1658	3.73 (3.23, 4.32) ***	3.79 (3.24, 4.44) ***	3.82 (3.26, 2.80) ***
Per 0.1 increment		1.50 (1.44, 1.56) ***	1.50 (1.44, 1.57) ***	1.50 (1.44, 1.57) ***
Stroke				
Robust	91/8791	Reference	Reference	Reference
Pre-frailty	176/5844	2.89 (2.23, 3.75) ***	2.56 (1.96, 3.34) ***	2.55 (1.96, 3.34) ***
Frailty	157/1658	8.96 (6.76, 11.87) ***	7.11 (5.29, 9.56) ***	7.04 (5.24, 9.47) ***
Per 0.1 increment		1.82 (1.71, 1.94) ***	1.71 (1.60, 1.83) ***	1.71 (1.59, 1.83) ***

Statistical significant at *$P < 0.05$, **$P < 0.01$, and ***$P < 0.001$.

Model 1 was adjusted age and gender.

Model 2 was additionally adjusted educational level, marital status, residence, smoking status, drinking status, hypertension, diabetes mellitus, dyslipidemia, and use of antihypertensive medications, antidiabetic medications and lipid-lowering therapy.

Model 3 was additionally adjusted BMI, SBP and DBP.

Table S4. Cross-sectional association between the frailty index and CVD in subpopulations of 10,803 participants underwent biomarkers measurements.

	OR (95% CI)		
	CVD	Heart disease	Stroke
Pre-frailty			
Model 3	2.54 (2.28, 2.84) ***	2.49 (2.22, 2.80) ***	2.55 (1.96, 3.34) ***
Model 3 plus			
FBG	2.51 (2.19, 2.87) ***	2.39 (2.07, 2.75) ***	2.85 (2.04, 3.98) ***
HbA1c	2.51 (2.19, 2.87) ***	2.39 (2.08, 2.75) ***	2.86 (2.05, 3.99) ***
TC	2.51 (2.19, 2.87) ***	2.39 (2.08, 2.75) ***	2.86 (2.05, 4.00) ***
TG	2.52 (2.20, 2.88) ***	2.39 (2.08, 2.76) ***	2.87 (2.05, 4.01) ***
LDL-c	2.51 (2.19, 2.88) ***	2.39 (2.08, 2.76) ***	2.87 (2.05, 4.00) ***
HDL-c	2.51 (2.19, 2.88) ***	2.39 (2.08, 2.75) ***	2.90 (2.07, 4.04) ***
eGFR	2.51 (2.20, 2.87) ***	2.39 (2.08, 2.76) ***	2.86 (2.05, 3.99) ***
All biomarkers	2.54 (2.21, 2.90) ***	2.41 (2.10, 2.78) ***	2.89 (2.07, 4.03) ***
Frailty			
Model 3	4.76 (4.10, 5.52) ***	3.82 (3.26, 2.80) ***	7.04 (5.24, 9.47) ***
Model 3 plus			
FBG	4.89 (4.09, 5.84) ***	3.78 (3.13, 4.57) ***	7.90 (5.47, 11.39) ***
HbA1c	4.89 (4.09, 5.84) ***	3.78 (3.13, 4.57) ***	7.90 (5.48, 11.40) ***
TC	4.90 (4.10, 5.86) ***	3.79 (3.14, 4.57) ***	7.93 (5.49, 11.43) ***
TG	4.90 (4.09, 5.84) ***	3.78 (3.13, 4.57) ***	7.90 (5.48, 11.39) ***
LDL-c	4.91 (4.11, 5.87) ***	3.79 (3.14, 4.58) ***	7.95 (5.51, 11.47) ***
HDL-c	4.89 (4.09, 5.84) ***	3.78 (3.13, 4.57) ***	7.90 (5.48, 11.40) ***
eGFR	4.92 (4.11, 5.88) ***	3.79 (3.14, 4.58) ***	7.87 (5.45, 11.35) ***
All biomarkers	4.95 (4.14, 5.92) ***	3.81 (3.16, 4.61) ***	7.92 (5.49, 11.44) ***
Per 0.1 increment			
Model 3	1.64 (1.57, 1.71) ***	1.50 (1.44, 1.57) ***	1.71 (1.59, 1.83) ***
Model 3 plus			
FBG	1.65 (1.56, 1.73) ***	1.50 (1.42, 1.58) ***	1.73 (1.59, 1.88) ***
HbA1c	1.65 (1.56, 1.73) ***	1.50 (1.42, 1.58) ***	1.73 (1.59, 1.89) ***
TC	1.65 (1.56, 1.74) ***	1.50 (1.42, 1.58) ***	1.74 (1.60, 1.89) ***
TG	1.64 (1.56, 1.73) ***	1.50 (1.42, 1.58) ***	1.73 (1.59, 1.88) ***
LDL-c	1.65 (1.56, 1.74) ***	1.50 (1.42, 1.58) ***	1.74 (1.60, 1.89) ***
HDL-c	1.64 (1.56, 1.73) ***	1.50 (1.42, 1.58) ***	1.73 (1.59, 1.88) ***
eGFR	1.65 (1.57, 1.74) ***	1.50 (1.42, 1.58) ***	1.74 (1.60, 1.89) ***
All biomarkers	1.66 (1.57, 1.74) ***	1.50 (1.42, 1.58) ***	1.74 (1.60, 1.90) ***

Statistically significant at **P* < 0.05, ***P* < 0.01, and ****P* < 0.001.

Abbreviation: FBG, fasting blood glucose; HbA1c, glycated hemoglobin; TC, total cholesterol; TG, triglycerides. LDL-c, low-density lipoprotein cholesterol; HDL-c, high-density lipoprotein cholesterol; eGFR, estimated glomerular filtration rate.

Model 3 was adjusted age, gender, educational level, marital status, residence, smoking status, drinking status, hypertension, diabetes mellitus, dyslipidemia, use of antihypertensive medications, antidiabetic medications and lipid-lowering therapy, BMI, SBP, DBP.

Table S5. Longitudinal association between the frailty index and CVD events in subpopulations of 8971 participants underwent biomarkers measurements.

	HR (95% CI)		
	CVD	Heart disease	Stroke
Pre-frailty			
Model 3	1.53 (1.39, 1.68) ***	1.62 (1.45, 1.81) ***	1.23 (1.03, 1.46) *
Model 3 plus			
FBG	1.54 (1.38, 1.72) ***	1.63 (1.44, 1.85) ***	1.24 (1.02, 1.52) *
HbA1c	1.54 (1.38, 1.72) ***	1.63 (1.44, 1.85) ***	1.24 (1.02, 1.51) *
TC	1.54 (1.38, 1.72) ***	1.63 (1.44, 1.85) ***	1.24 (1.02, 1.52) *
TG	1.54 (1.38, 1.72) ***	1.63 (1.44, 1.85) ***	1.25 (1.02, 1.52) *
LDL-c	1.54 (1.38, 1.72) ***	1.64 (1.44, 1.86) ***	1.24 (1.02, 1.52) *
HDL-c	1.54 (1.38, 1.72) ***	1.63 (1.44, 1.85) ***	1.25 (1.02, 1.53) *
eGFR	1.54 (1.38, 1.72) ***	1.63 (1.44, 1.85) ***	1.24 (1.02, 1.51) *
All biomarkers	1.54 (1.38, 1.72) ***	1.63 (1.44, 1.85) ***	1.26 (1.03, 1.53) *
Frailty			
Model 3	2.17 (1.88, 2.50) ***	2.16 (1.83, 2.56) ***	2.06 (1.63, 2.63) ***
Model 3 plus			
FBG	2.11 (1.79, 2.49) ***	2.19 (1.81, 2.64) ***	1.97 (1.48, 2.61) ***
HbA1c	2.11 (1.79, 2.49) ***	2.19 (1.81, 2.64) ***	1.98 (1.49, 2.62) ***
TC	2.12 (1.80, 2.50) ***	2.19 (1.81, 2.65) ***	1.99 (1.50, 2.64) ***
TG	2.11 (1.79, 2.49) ***	2.19 (1.81, 2.64) ***	1.98 (1.49, 2.62) ***
LDL-c	2.11 (1.79, 2.49) ***	2.20 (1.82, 2.66) ***	1.99 (1.50, 2.64) ***
HDL-c	2.11 (1.79, 2.49) ***	2.19 (1.81, 2.64) ***	1.97 (1.48, 2.61) ***
eGFR	2.11 (1.78, 2.49) ***	2.18 (1.80, 2.64) ***	1.98 (1.49, 2.62) ***
All biomarkers	2.12 (1.80, 2.50) ***	2.20 (1.82, 2.66) ***	1.99 (1.50, 2.64) ***
Per 0.1 increment			
Model 3	1.29 (1.24, 1.34) ***	1.28 (1.22, 1.34) ***	1.28 (1.03, 1.46) ***
Model 3 plus			
FBG	1.29 (1.23, 1.35) ***	1.29 (1.22, 1.35) ***	1.29 (1.19, 1.40) ***
HbA1c	1.29 (1.23, 1.35) ***	1.28 (1.22, 1.35) ***	1.29 (1.19, 1.40) ***
TC	1.29 (1.23, 1.35) ***	1.28 (1.22, 1.35) ***	1.29 (1.19, 1.40) ***
TG	1.29 (1.23, 1.35) ***	1.28 (1.22, 1.35) ***	1.29 (1.19, 1.40) ***
LDL-c	1.29 (1.23, 1.35) ***	1.29 (1.22, 1.35) ***	1.29 (1.19, 1.40) ***
HDL-c	1.29 (1.23, 1.35) ***	1.28 (1.22, 1.35) ***	1.29 (1.19, 1.40) ***
eGFR	1.29 (1.23, 1.35) ***	1.28 (1.22, 1.35) ***	1.29 (1.19, 1.40) ***
All biomarkers	1.29 (1.23, 1.35) ***	1.28 (1.22, 1.35) ***	1.29 (1.19, 1.40) ***

Statistically significant at *$P < 0.05$, **$P < 0.01$, and ***$P < 0.001$.

Abbreviation: FBG, fasting blood glucose; HbA1c, glycated hemoglobin; TC, total cholesterol; TG, triglycerides. LDL-c, low-density lipoprotein cholesterol; HDL-c, high-density lipoprotein cholesterol; eGFR, estimated glomerular filtration rate.

Model 3 was adjusted age, gender, educational level, marital status, residence, smoking status, drinking status, hypertension, diabetes mellitus, dyslipidemia, use of antihypertensive medications, antidiabetic medications and lipid-lowering therapy, BMI, SBP, DBP.

Table S6. Longitudinal association between the frailty index and CVD events by competing risk analysis

Outcome	Case/N	Incidence per 1000 person-years	Model 1 HR (95% CI)	Model 2 HR (95% CI)	Model 3 HR (95% CI)
CVD events					
Robust	954/7917	22.47	Reference	Reference	Reference
Pre-frailty	890/4583	36.52	1.51 (1.37, 1.65) ***	1.49 (1.35, 1.63) ***	1.51 (1.37, 1.65) ***
Frailty	278/1080	62.21	1.96 (1.70, 2.26) ***	1.91 (1.65, 2.20) ***	1.95 (1.79, 2.25) ***
Per 0.1 increment			1.24 (1.20, 1.29) ***	1.23 (1.18, 1.27) ***	1.23 (1.19, 1.28) ***
Heart disease					
Robust	707/7917	16.43	Reference	Reference	Reference
Pre-frailty	701/4583	28.30	1.59 (1.43, 1.77) ***	1.58 (1.42, 1.76) ***	1.60 (1.43, 1.78) ***
Frailty	209/1080	41.60	1.95 (1.66, 2.30) ***	1.93 (1.64, 2.28) ***	1.97 (1.67, 2.33) ***
Per 0.1 increment			1.23 (1.19, 1.29) ***	1.22 (1.18, 1.28) ***	1.23 (1.18, 1.28) ***
Stroke					
Robust	322/7917	7.25	Reference	Reference	Reference
Pre-frailty	256/4583	9.71	1.25 (1.05, 1.47) **	1.18 (0.99, 1.40)	1.20 (1.02, 1.43) *
Frailty	102/1080	18.38	1.97 (1.55, 2.52) ***	1.77 (1.38, 2.27) ***	1.81 (1.41, 2.33) ***
Per 0.1 increment			1.24 (1.16, 1.33) ***	1.20 (1.12, 1.29) ***	1.21 (1.13, 1.30) ***

Statistically significant at **P* < 0.05, ***P* < 0.01, and ****P* < 0.001.

Model 1 was adjusted age and gender.

Model 2 was additionally adjusted educational level, marital status, residence, smoking status, drinking status, hypertension, diabetes mellitus, dyslipidemia, and use of antihypertensive medications, antidiabetic medications and lipid-lowering therapy.

Model 3 was additionally adjusted BMI, SBP and DBP.

Table S7. Cross-sectional association between the frailty index and CVD in complete dataset

	Model 1		Model 2		Model 3	
	Case/N	OR (95% CI)	Case/N	OR (95% CI)	Case/N	OR (95% CI)
CVD						
Robust	688/8791	Reference	673/8672	Reference	505/6619	Reference
Pre-frail	1093/5844	2.47 (2.23, 2.75) ***	1075/5768	2.54 (2.27, 2.84) ***	848/4807	2.44 (2.15, 2.77) ***
Frail	537/1658	4.64 (4.05, 5.32) ***	525/1610	4.81 (4.14, 5.58) ***	381/1212	4.86 (4.096, 5.78) ***
Per 0.1 increment		1.63 (1.57, 1.69) ***		1.64 (1.57, 1.72) ***		1.75 (1.66, 1.85) ***
Heart disease						
Robust	611/8791	Reference	598/8672	Reference	447/6619	Reference
Pre-frail	965/5844	2.40 (2.15, 2.68) ***	949/5768	2.49 (2.21, 2.80) ***	741/4807	2.38 (2.09, 2.72) ***
Frail	421/1658	3.73 (3.23, 4.32) ***	412/1610	3.84 (3.28, 4.50) ***	304/1212	4.06 (3.38, 4.87) ***
Per 0.1 increment		1.50 (1.44, 1.56) ***		1.50 (1.44, 1.57) ***		1.62 (1.54, 1.71) ***
Stroke						
Robust	91/8791	Reference	89/8672	Reference	68/6619	Reference
Pre-frail	176/5844	2.89 (2.23, 3.75) ***	174/5768	2.59 (1.98, 3.39) ***	143/4807	2.54 (1.88, 3.44) ***
Frail	157/1658	8.96 (6.76, 11.87) ***	153/1610	7.23 (5.38, 9.78) ***	102/1212	6.43 (4.55, 9.09) ***
Per 0.1 increment		1.82 (1.71, 1.94) ***		1.73 (1.61, 1.85) ***		1.80 (1.65, 1.98) ***

Statistically significant at $^{*}P < 0.05$, $^{**}P < 0.01$, and $^{***}P < 0.001$.

Model 1 was adjusted age and gender.

Model 2 was additionally adjusted educational level, marital status, residence, smoking status, drinking status, hypertension, diabetes mellitus, dyslipidemia, and use of antihypertensive medications, antidiabetic medications and lipid-lowering therapy.

Model 3 was additionally adjusted BMI, SBP, DBP.

Table S8. Longitudinal association between the frailty index and CVD events in complete dataset

	Model 1		Model 2		Model 3	
	Case/N	HR (95% CI)	Case/N	HR (95% CI)	Case/N	HR (95% CI)
CVD						
Robust	954/7917	Reference	949/7817	Reference	747/5978	Reference
Pre-frail	890/4583	1.53 (1.39, 1.68) ***	883/4531	1.51 (1.37, 1.66) ***	760/3827	1.54 (1.39, 1.71) ***
Frail	278/1045	2.19 (1.91, 2.52) ***	275/1045	2.15 (1.86, 2.48) ***	217/804	2.22 (1.89, 2.60) ***
Per 0.1 increment		1.30 (1.25, 1.35) ***		1.28 (1.23, 1.33) ***		1.34 (1.28, 1.41) ***
Heart disease						
Robust	707/7917	Reference	702/7817	Reference	551/5978	Reference
Pre-frail	791/4583	1.61 (1.44, 1.79) ***	694/4531	1.60 (1.44, 1.79) ***	595/3827	1.63 (1.45, 1.84) ***
Frail	209/1045	2.14 (1.82, 2.52) ***	207/1045	2.14 (1.82, 2.53) ***	171/804	2.32 (1.94, 2.79) ***
Per 0.1 increment		1.28 (1.23, 1.34) ***		1.27 (1.22, 1.33) ***		1.36 (1.28, 1.43) ***
Stroke						
Robust	322/7917	Reference	322/7817	Reference	255/5978	Reference
Pre-frail	256/4583	1.27 (1.07, 1.50) *	255/4531	1.20 (1.01, 1.43) *	218/3827	1.21 (1.01, 1.48) *
Frail	102/1045	2.26 (1.79, 2.86) ***	100/1045	2.02 (1.58, 2.57) ***	71/804	1.85 (1.40, 2.45) ***
Per 0.1 increment		1.31 (1.23, 1.41) ***		1.26 (1.18, 1.36) ***		1.26 (1.15, 1.38) ***

Statistically significant at $^{*}P < 0.05$, $^{**}P < 0.01$, and $^{***}P < 0.001$.

Model 1 was adjusted age and gender.

Model 2 was additionally adjusted educational level, marital status, residence, smoking status, drinking status, hypertension, diabetes mellitus, dyslipidemia, and use of antihypertensive medications, antidiabetic medications and lipid-lowering therapy.

Model 3 was additionally adjusted BMI, SBP, DBP.

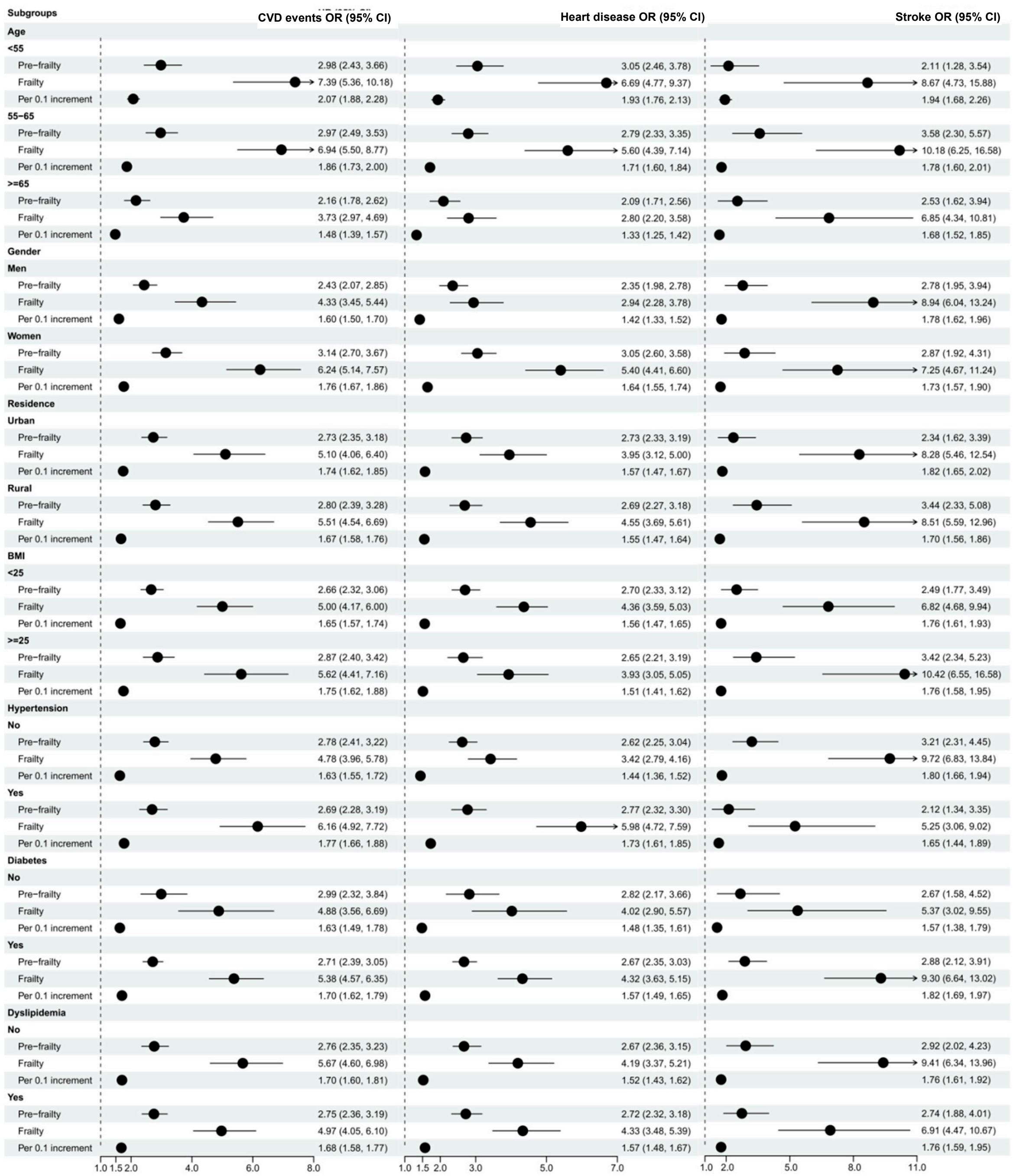

Figure S1. Cross-sectional association between the frailty index and CVD stratified by age, gender, residence, BMI, and chronic disease status

Model was adjusted for age, gender, educational level, marital status, residence, smoking status, drinking status, hypertension, diabetes mellitus, dyslipidemia and BMI.
